# The perceptions of general practice among Central and Eastern Europeans in the United Kingdom: A systematic scoping review

**DOI:** 10.1111/hex.13433

**Published:** 2022-01-19

**Authors:** Aaron Poppleton, Kelly Howells, Isabel Adeyemi, Carolyn Chew‐Graham, Lisa Dikomitis, Caroline Sanders

**Affiliations:** ^1^ Centre for Primary Care and Health Services Research University of Manchester Manchester UK; ^2^ School of Medicine Keele University Keele UK; ^3^ NIHR Greater Manchester Patient Safety Translational Research Centre (GM‐PSTRC) Manchester UK; ^4^ Kent and Medway Medical School University of Kent and Canterbury Christ Church University Canterbury UK

**Keywords:** delivery of healthcare, emigration immigration, European Union, general practice, United Kingdom

## Abstract

**Background:**

Around 2 million people have migrated from Central and Eastern Europe to the UK since 2004. The UK Central and Eastern European Community (UK‐CEE) are disproportionately exposed to the social determinants of poor physical and mental health. Their health and healthcare beliefs remain under‐researched, particularly regarding primary care.

**Objective:**

This review explores UK‐CEE community members' use and perceptions of UK general practice.

**Methods:**

A systematic search of nine bibliographic databases identified 2094 publications that fulfilled the search criteria. Grey literature searches identified 16 additional relevant publications. Screening by title and abstract identified 201 publications of relevance, decreasing to 65 after full‐text screening. Publications were critically appraised, with data extracted and coded. Thematic analysis using constant comparison allowed generation of higher‐order thematic constructs.

**Results:**

Full UK‐CEE national representation was achieved. Comparatively low levels of GP registration were described, with ability, desire and need to engage with GP services shaped by the interconnected nature of individual community members' cultural and sociodemographic factors. Difficulties overcoming access and in‐consultation barriers are common, with health expectations frequently unmet. Distrust and dissatisfaction with general practice often persist, promoting alternative health‐seeking approaches including transnational healthcare. Marginalized UK‐CEE community subgroups including Roma, trafficked and homeless individuals have particularly poor GP engagement and outcomes. Limited data on the impact of Brexit and COVID‐19 could be identified.

**Conclusions:**

Review findings demonstrate the need for codesigned approaches to remove barriers to engagement, culturally adapt and develop trust in GP care for UK‐CEE individuals.

**Community Involvement:**

Community members and stakeholders shaped the conceptualisation of the review question and validation of emergent themes.

## 1 INTRODUCTION

The past century has witnessed unprecedented global population migration.^1^ Migrants are typically younger and physically fit individuals.^2^, ^3^ Longitudinal exposure to socially determined risks factors for poor health can erode this health premium.^4^, ^5^, ^6^, ^7^, ^8^, ^9^, ^10^ The United Kingdom provides health coverage for all permanent residents and certain economic migrants.^11^ UK healthcare providers' awareness of migrants' motivations and challenges in using healthcare services varies, affecting the degree of inclusion of migrant perspectives in service design,^12^, ^13^, ^14^, ^15^ communication^16^, ^17^, ^18^ and levels of general practice registration.^19^

### 1.1 Specific healthcare needs of UK Central and Eastern European migrants

Facilitated by simplified migration within the European Union (EU), approximately 2 million Central and Eastern Europeans (CEEs) have migrated to the United Kingdom since 2004,^20^ continuing throughout the implementation of Brexit.^21^ CEEs within the UK (UK‐CEE) are heterogeneous in terms of nationality, language, age and socioeconomic status. They are, however, united by shared home nation political and socioeconomic histories, alongside migration and integration experiences within UK society.^22^ Similar health system reforms across CEE counties over the past 30 years have included the introduction of public health insurance, greater emphasis on primary healthcare (including general practice), increased formal and informal out‐of‐pocket healthcare payments and market entry of privately owned outpatient specialist clinics.^23^

UK census and healthcare data classify CEEs as ‘White Other’.^24^, ^25^ In contrast to many other ‘white’ migrant communities, there is evidence of poor physical and mental health outcomes at a locality level, particularly for common mental health disorders including anxiety, suicide and alcohol overuse.^26^, ^27^, ^28^, ^29^ A review of UK‐CEE healthcare access found commonalities in dissatisfaction with UK healthcare, stemming from language barriers and a mismatch between healthcare expectations and service provision.^30^ Dissatisfaction with GP care has been cited as a reason for low UK‐CEE rates of GP registration and inappropriate emergency department  (ED) use.^30^, ^31^ Patterns of primary care and private healthcare service use may also be influenced by shared UK‐CEE help‐seeking behaviours, service knowledge and perceptions of GP accessibility.^16^, ^32^, ^33^, ^34^

To date, no systematic review has explored the frequency and variability in factors influencing UK‐CEEs' registration, use and perceptions of UK general practice. As such, it is unclear where research gaps exist, including the particular needs and experiences of people from distinct countries within our definition of CEE. Primary care staffs' cultural competency towards UK‐CEEs has also received limited attention.^34^, ^35^ As EU citizens, the Brexit referendum, negotiation process (2016–2020) and subsequent transition period have changed the perceived and actual legal position, rights and healthcare costs for CEE citizens in the United Kingdom. The COVID‐19 pandemic has further potential to influence UK‐CEEs' access and experience of UK general practice.^36^, ^37^ Characterization of these factors is required to develop strategies to overcome barriers to engagement and improve satisfaction with general practice.^38^, ^39^

Scoping reviews increase the representation of community knowledge, allowing exploration and mapping of the extent, range and nature of scientific literature that may not otherwise be identified.^40^, ^41^ This summary of knowledge is of benefit to policymakers, stakeholders and clinicians, informing a timely response to identified health concerns.^42^ We aim to provide a longitudinal review of UK‐CEE individuals' perceptions and engagement with general practice.

### 1.2 Community consultation

The first author met with community groups, organisations and researchers working with UK‐CEEs in North West England (2016–2020). Community members expressed repeated difficulties and frustration with general practice in the United Kingdom. Community organisations described a paucity of high‐quality research on UK‐CEE health beliefs and general practice engagement but knew of local assessments and reports. Outcomes from community consultation informed the initial conceptualisation and ongoing development of the review.

## 2 METHODS

A systematic literature search was undertaken using established scoping review methodology.^40^, ^43^, ^44^ The search strategy comprised (1) defining the research question; (2) identification of relevant research publications; (3) data abstraction and charting; (4) summary, synthesis and reporting of results; and (5) stakeholder consultation.

### 2.1 Defining the research question

The primary review aim was to identify ‘what are UK resident CEE nationals' perceptions of, and engagement with UK General Practice?’ A number of subquestions were also explored, namely, (1) whether barriers and facilitators exist for UK‐CEE community member engagement with UK general practice; (2) whether any identified barriers and/or facilitators vary in their degree and scope for different members of the UK‐CEE community according to gender, age, socioeconomic status, ethnic affiliation or other individual characteristics; and (3) how these barriers and/or facilitators vary over time for the UK‐CEE community, including ‘Brexit’. For the purposes of this review, search terms relating to the UK‐CEE community were defined as nationals from EU member nations in Central or Eastern Europe (A8/A2 nations and Croatia [Table 1]) who migrated to the United Kingdom from 2004.^45^

**Table 1:** Accession dates of Central and Eastern European nations to the European Union 2004–2013

Year	Nations
2004	A8: Czech Republic, Estonia, Hungary, Latvia, Lithuania, Poland, Slovakia, Slovenia
2007	A2: Bulgaria, Romania
2013	Croatia

### 2.2 Search strategy

We identified four core question concepts, relating to the population (Central and/or Eastern European; Migration), location (United Kingdom) and ‘intervention’ (General Practice). Relevant search terms were identified from Medical Subject Heading codes and peer‐reviewed publications.^46^, ^47^ Nine medical bibliographic databases were searched (Applied Social Sciences Index Abstracts; Cumulative Index to Nursing and Allied Health Literature; Embase; International Bibliography of the Social Sciences; OvidMEDLINE; PsycInfo; Scopus; Sociological abstracts; and Web of Science). Single‐item searches were combined through Boolean operators to develop concept‐wide searches. Discussion with a review specialist led to increased returns through the addition of broader healthcare‐related terms (Appendix SA). Lastly, concept searches were combined.

Supplemental grey literature and publication reference list searches were undertaken.^48^, ^49^, ^50^ Academic consensus on the definition, search strategy and visibility of grey literature has yet to be reached.^49^, ^50^, ^51^, ^52^, ^53^ Researcher, librarian and community organisation recommendations informed criteria for literature formats (Primary data within: conference proceedings, journal and magazine articles, academic dissertations, institutional and organisational reports and book chapters)^49^ and search sources (online databases, search engines, repositories (university, institutional) and library catalogues) (Appendix SB). Systematic searches were guided by the Canadian Agency for Drugs and Technologies in Health GreyMatters Tool^54^ and simple searches incorporating search terms from each category.^48^, ^52^ Peer and grey literature searches were undertaken concurrently. The included publications are presented in Appendix SC.

### 2.3 Inclusion criteria

Inclusion and exclusion criteria are presented (Table 2), with justification (Table 3). The accepted publication date range was May 2004–July 2020. To maximize conceptual coverage, all qualitative and quantitative research containing primary data were considered for inclusion.^40^

**Table 2:** Scoping review inclusion and exclusion criteria

Inclusion criteria	Exclusion criteria
− English language − Published from 1 May 2004 onwards − Provide information on adult Central and Eastern European citizens' usage of engagement with UK general practice	− Non‐English‐language publications − Studies falling outside of the review aim (e.g., not relevant to UK primary care)

**Table 3:** Justification of review eligibility criteria

Time frame	The time frame chosen aimed to capture studies conducted since EU enlargement to incorporate the Central and Eastern European nations, and resultant freedom of movement to live and work in the United Kingdom
Language	Non‐English‐language publications were excluded based on the time and costs required for translation
Age	The review was limited to adult community members to improve comparability in the studies considered

Abbreviation: EU, European Union.

### 2.4 Data abstraction and charting

**Figure 1:**
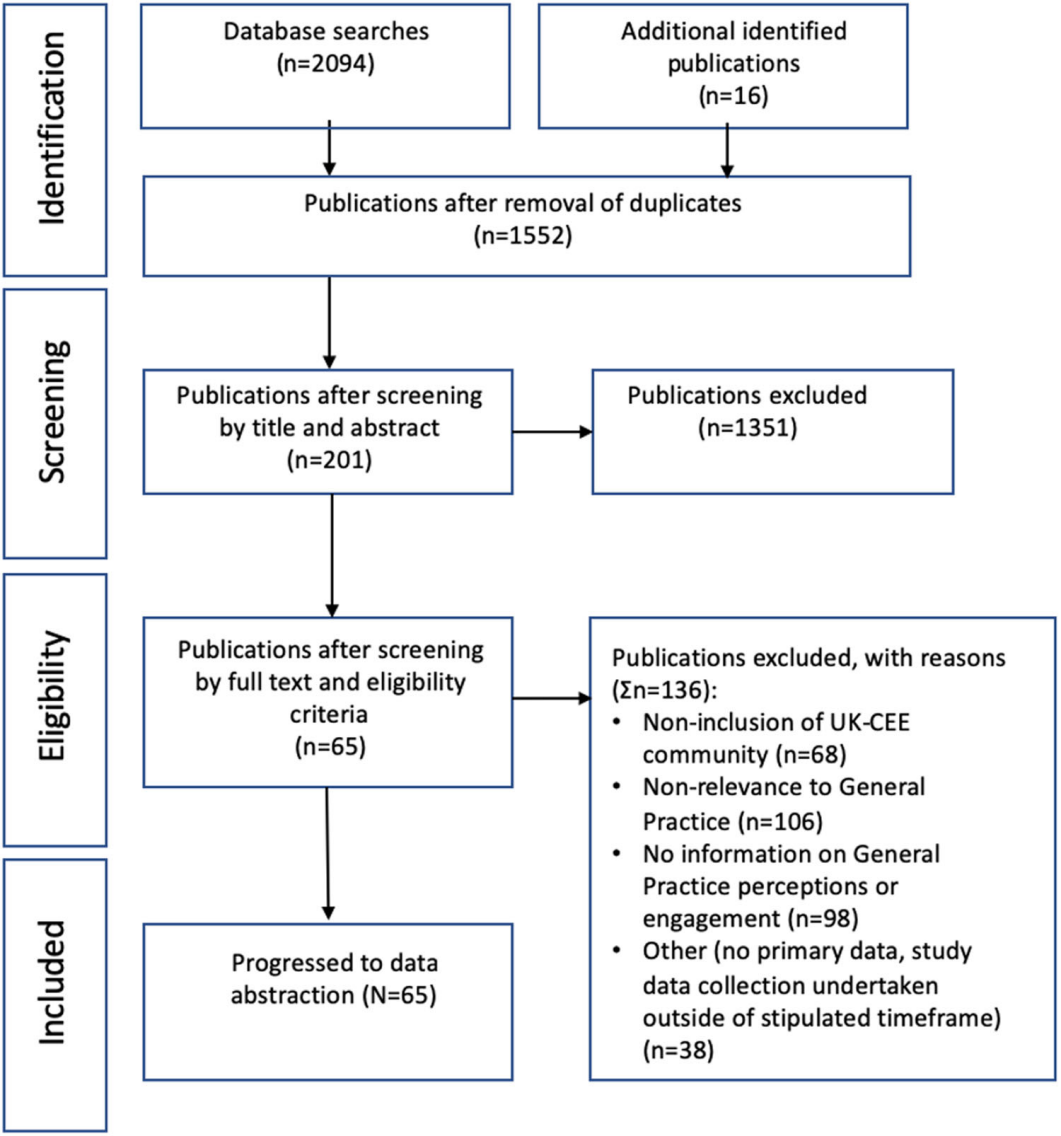
PRISMA chart displaying the identification, screening, eligibility and inclusion of publications

Database search results were exported to Endnote (version 9.1), merged and deduplicated. In keeping with PRISMA guidelines, search results were screened over two stages (Figure 1).^55^

#### 2.4.1 Stage 1: Title and abstract screening

Publication titles and abstracts were screened for eligibility and general relevance. General relevance constituted (1) focus on Central and/or Eastern Europeans; (2) healthcare use; and (3) UK context. Full‐text review was undertaken if all criteria were fulfilled or in cases of uncertainty.

#### 2.4.2 Stage 2: Full‐text screening

We rechecked the inclusion and exclusion criteria, and assessed relevance to the primary aim. Articles had to fulfil the following inclusion criteria:

1. Key term/s (≥1): ‘general practice’; ‘GP’; ‘family medicine’; ‘family practice’; ‘primary care’; or ‘doctor’ (in the context of community healthcare services).
2. Participants: Central European, Eastern European, A8 or A2 nation migrants.
3. Perceptions and/or engagement with healthcare.

Publications focussing solely on European migrants not from Central Europe, Eastern Europe or non‐A8/A2 nations where individuals identified as being from Central and Eastern Europe, or where outcomes relating to UK‐CEEs could not be clearly differentiated, were excluded. Uncertainties regarding study inclusion were cross‐checked by three primary care researchers and resolved through discussion with arbitration by a senior academic. Reasons for noninclusion were documented. Given the nature of the review, statistical inter‐rater agreement was not calculated.^56^

#### 2.4.3 Quality assessment

Publication quality does not typically influence scoping review inclusion or weighting.^40^, ^57^ Critical appraisal was undertaken using design‐specific quality assessment checklists to aid interpretation, with colour‐coded numerical scoring indicating the degree of fulfilment (Appendix SD).^58^ Publication quality was generally good, with included publications having high (*n* = 50), moderate (*n* = 12) or low (*n* = 3) critical appraisal scores.

A data abstraction chart was developed, piloted on three shortlisted publications, discussed within the team and refined. Concise text‐based information was extracted from included publications and uploaded to NVivo.^59^

### 2.5 Summary, synthesis and reporting of results

A representative sample of three transcripts was reread and coded independently by team members. Discussion of the emerging codes formed the basis of a coding scheme for the remaining transcripts. Tabulated coded data were used to explore relationships between study outcomes, enabling inductive and iterative generation of emergent themes, followed by thematic analysis with constant comparison, rather than prior theory, to clarify higher‐order constructs.^60^ Specific attention was paid to the applicability of findings to population subgroups.^61^ The lead author discussed emergent review themes with individuals from Poland, Lithuania, Romania and UK‐CEE community organisations in person and by phone, including Roma and homeless individuals. Review findings were felt to broadly agree with individuals' and organisations' experiences of engagement with GP services including clinician contact, prescribing practises and service dissatisfaction.

## 3 RESULTS

### 3.1 Search results

Database searches identified 2094 publications, decreasing to 1536 after deduplication. Title and abstract screening identified 185 publications that fully (77) or partially (108) fulfilled criteria for full‐text review. Forty‐nine publications fulfilled the criteria for data abstraction. Additional and grey literature searches identified 16 further publications (Figure 1).

### 3.2 Study characteristics

#### 3.2.1 Outcome measures

The most commonly identified study forms were peer‐reviewed research publications (*n* = 45) and local government or third‐sector organisation reports (*n* = 11), with smaller numbers of academic theses, conference abstracts, magazine articles, oral presentation summaries and letters also fulfilling inclusion criteria (Table 4). Study methodologies included qualitative (*n* = 28), quantitative (*n* = 10), mixed methods (qualitative and quantitative) and smaller numbers of case reports, case series, audits and other study designs (Table 5). Publication data collection range being before (*n* = 46), during (*n* = 5) and from (*n* = 14) the 2016 Brexit referendum (Table 6).

**Table 4:** Publications fulfilling inclusion criteria listed by type

Publication type	Publications (*n*)	Percentage (%)
Research paper	45	69.2%
Report	11	16.9%
Thesis	3	4.6%
Abstract	2	3.1%
Magazine article	2	3.1%
Oral presentation	1	1.5%
Letter	1	1.5%
Total	65	

**Table 5:** Selected publications listed by study design

Study design	Publications (*n*)	Percentage (%)
Qualitative methods (of which mixed qualitative methods)	28 (6)	43.0% (9.2%)
Quantitative methods	10	15.4%
Mixed methods (qualitative and quantitative)	9	13.8%
Case report	3	4.6%
Case series	2	3.1%
Audit	2	3.1%
Other (epidemiological profile, service evaluation, health economics, policy document, record linkage study)	10	15.4%
Total	65	

**Table 6:** Selected publications listed by data of data collection

Year of data collection	Publications (*n*)	Percentage (%)
2004–2015	46	70.8%
2016–2020	14	21.5%
2004–2015 and 2016–2020	5	7.7%
Total	65	

#### 3.2.2 Participant demographics

All A8 and A2 UK‐CEE nationalities were described (Table 7). Polish (*n* = 42), Slovak (*n* = 18), UK‐CEE (nonspecified) (*n* = 14) and Romanian (*n* = 13) were the most commonly described nationalities. Publications frequently considered multiple UK‐CEE nationalities simultaneously and as a single entity, rather than comparatively. Several studies either additionally or exclusively considered the experiences and perceptions of healthcare and community workers working with the UK‐CEEs (*n* = 8). Publications drew on data from across the United Kingdom (Table 8), including England (*n* = 42) and Scotland (*n* = 13). Within England, (*n* = 13) studies took place in London. Wales and Northern Ireland were less well represented. The setting varied, incorporating urban (*n* = 27), smaller towns or rural (*n* = 11) and mixed settings (*n* = 10; Table 8).

**Table 7:** UK‐CEE nationality coverage within studies

	Publication inclusion (*n*)	Percentage (%)
Polish (*of which Roma*)	42 (1)	64.6% (1.5%)
Slovakian (*of which Roma*)	18 (6)	27.7% (9.2%)
Central and Eastern European or A8/A2 nationality (not‐specified)	14	21.5%
Romanian (*of which Roma*)	13 (5)	20.0% (7.7%)
Lithuanian	13	20.0%
Czech (*of which Roma*)	10 (1)	15.4% (1.5%)
Bulgarian (*of which Roma*)	7 (1)	10.8% (1.5%)
Latvian	7	10.8%
Hungarian	5	7.7%
Slovenian	3	4.6%
Estonian	2	3.1%
Noncentral and Eastern Europeans (healthcare and community workers)	8	12.3%

**Table 8:** Location of study data collection by nation and setting

	Publication inclusion (*n*)	Percentage (%)
England (of which London)	42 (13)	64.6% (20.0%)
Scotland	13	20.0%
Wales	1	1.5%
Northern Ireland	4	6.2%
UK—not stated	7	10.8%
Other location	2	3.1%
Urban (large city)	27	41.5%
Town or rural	11	16.9%
Mixed setting (urban/town/rural)	10	15.4%
Setting—not stated	17	26.2%
Urban (large city)	27	41.5%

#### 3.2.3 Emergent themes

**Figure 2:**
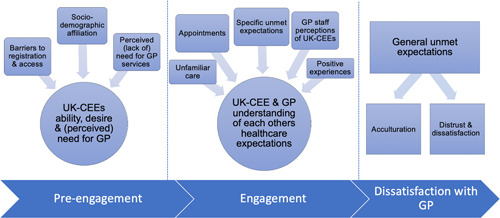
UK resident Central and Eastern Europeans' perceptions of and engagement with general practice in the United Kingdom

Three emergent themes were identified based on UK‐CEEs' degree of engagement with general practice services, their encounters with general practice services and the impact of unmet expectations on future general practice engagement (Figure 2).

### 3.3 Theme 1: General practice engagement is shaped by UK‐CEE community members' ability, desire and need to engage with UK healthcare

Rates of general practice registration ranged between 12%^62^ and 87.9%,^63^ with no significant variation able to be discerned between the devolved UK nations. At an individual level, UK‐CEE community members' ability, desire and perceived or actual need to engage with general practice and navigate facilitators or barriers were shaped by an interaction of personal, social and cultural factors (Table 9).^32^, ^33^, ^39^, ^64^, ^65^, ^66^, ^67^ These coalesced into three broad, partially overlapping, cross‐nationality groupings:

1. The general UK‐CEE community, characterized by variable knowledge of UK GP services, deprioritized registration, barriers to engagement, unmet health expectations and dissatisfaction. Culturally familiar (transnational) healthcare frequently substituted or supplemented general practice use.^33^, ^62^, ^64^, ^68^, ^69^, ^70^, ^71^, ^72^
2. Young(er) individuals with higher educational, socioeconomic, social integration and English‐language status. Engagement and acceptance of GP care was comparatively higher.^33^, ^67^
3. Marginalized community subgroups with income and accommodation insecurity, including Roma, homeless, trafficked and unregistered individuals. High levels of (unmet) health needs were compounded by multifactorial barriers to general practice registration and engagement,^73^, ^74^, ^75^, ^76^, ^77^ including limited English or socially discouraged independent uptake, for example, female Roma or trafficked individuals.^77^, ^78^

**Table 9:** Factors increasing and decreasing the likelihood of individual UK‐CEE general practice registration and/or engagement

Factors increasing likelihood of registration and/or engagement	− Information on and/or support with registration − Supportive community networks in the United Kingdom − English‐language proficiency − Cultural integration − Employer requirement of GP registration − Intention to settle in the United Kingdom − Longer duration living in the United Kingdom − Married/cohabiting (particularly if with a non‐co‐national) − Family with children − Gender—female − Higher educational attainment
Factors decreasing the likelihood of registration and/or engagement	− Lack of health system knowledge − Poor English‐language skills − Limited community networks in the United Kingdom − Limited health literacy − Recent arrival to the United Kingdom − Short intended duration in the United Kingdom − Gender—male

#### 3.3.1 Structural barriers to registration

GP registration was not perceived as straightforward, with community members often lacking knowledge and guidance on the process and requirements, including personal documentation.^32^, ^67^, ^72^ In areas of recent migration, some practices were at full capacity, necessitating out of area registration.^79^, ^80^

The ability to gain and maintain practice registration was affected by uncertainty around healthcare entitlements, lack of clarity on GP role and accommodation transiency (UK and transnational).^80^ Transiency was most pronounced in those with unofficial residency,^33^, ^81^ casual or undocumented employment,^33^ Roma,^75^, ^77^ homeless^74^, ^76^ or trafficked individuals including sex‐workers,^73^ with reports of deregistration (without consent) due to missed appointments. The need to provide documentation raised concerns of UK authority awareness and subsequent deportation.^33^, ^67^, ^81^ Lack of a formal address risked care ‘charges to Overseas Visitors’.^76^ Lack of GP registration and/or service knowledge and perceived urgency were associated with higher ED use.^82^, ^83^ ED support in GP registration was viewed positively.^84^

#### 3.3.2 Structural barriers to general practice access

Frequently described and interconnected barriers to access and engagement with UK general practice included the following:

1. *Service understanding*: Limited awareness and understanding of general practice within the United Kingdom.^31^, ^33^, ^83^, ^85^, ^86^
2. *Limited English‐language ability*: Affecting understanding of healthcare correspondence, appointment booking and communication with clinicians.^31^, ^67^, ^83^, ^85^
3. *Medical information*: Limited availability or supply of non‐English‐language medical information^39^, ^67^ or interpreters.^16^, ^87^
4. *Appointment availability*: Both on inquiry and timing in light of other commitments, particularly work.^32^, ^39^, ^67^, ^71^, ^80^, ^88^

Structural barriers were reduced through informal co‐national support networks that provided service knowledge, encouraged healthcare review, supported GP registration and provided translation (in‐consultation, health correspondence, health information).^16^, ^27^, ^33^, ^67^, ^81^, ^89^ Peer disinformation, negative perceptions and use of transnational healthcare could, however, also normalize GP nonattendance, particularly where personal barriers to engagement already existed.^33^

#### 3.3.3 Increased perceived need for primary care services

An increased need for care was seen in:

1. *Non‐Polish individuals* and *those living away from urban centres* who had fewer culturally familiar health options.^33^, ^63^
2. *Individuals with children*, where more frequent contact stemmed from health visitor, immunisation and childhood illness appointments (which were perceived as urgent).^32^, ^33^, ^62^, ^65^, ^80^
3. *Limited finances* for example, individuals without social security coverage to access state or personal finances for private home nation healthcare.^33^
4. *Acute or perceived severe health needs*: Initial health engagement could be tortuous and have avoided general practice.^33^, ^65^, ^77^ Ongoing avoidance and fear of engagement led to some individuals dying from untreated conditions.^27^, ^33^
5. *Contraception and sexual health*: Uptake varied by locality, familiarity with GP services and degree of empowerment,^39^, ^64^, ^86^ with female Roma and sex workers having comparatively low levels of registration and uptake.^83^, ^90^, ^91^

#### 3.3.4 Lack or deprioritization of perceived GP need

In a number of instances, registration and use of GP services were delayed or not attempted due to a lack of desire or perceived need.^33^, ^62^, ^64^, ^68^, ^69^, ^70^, ^71^, ^72^ Deprioritization against more urgent life pressures was common for example, attainment of accommodation, employment (long or unsociable shift patterns) and financial stability (particularly in London).^33^, ^67^ A lack of perceived healthcare need was most common in younger adults, men, recent arrivals and those intending to stay in the United Kingdom for a short period.^27^, ^33^, ^62^, ^67^, ^68^, ^69^, ^70^, ^71^, ^81^ Perceived need for UK general practice was shaped by perceptions of self‐care and transnational healthcare use.^33^, ^90^ Reasons for UK GP attendance showed only partial overlap with healthcare attendance in an individual's nation of origin.^85^ Conditions deprioritized for GP attendance included:

1. *Mental health symptoms*, despite a high prevalence, rarely led to GP presentation.^27^, ^87^, ^92^ Previous help‐seeking, greater National Health Service (NHS) knowledge and poorer mental health increased the likelihood of attendance.^93^ Less than 25% attended a GP in the 6 months preceding suicide, often for physical concerns, including chronic disease.^27^ Relationships and social connectedness were protective mental health factors.^27^, ^93^
2. *Screening*. Accommodation transiency, cyclical migration and requirement for GP registration impacted upon receiving appointment and screening letters.^39^, ^46^, ^88^, ^94^ GP invitations for childhood immunisations, health checks, cervical, breast and colorectal screening, when received, understood and convenient, were commonly accepted.^39^, ^70^, ^89^ Understanding of screening indications was limited, with concerns around frequency, quality or inconvenience leading some to pursue additional screening in home nations.^39^, ^70^ Many personal barriers to screening attendance were similar to UK nationals.^39^
3. Health promotion. GP health promotion strategies were not actively sought out due to more urgent life pressures,^33^, ^70^ with associated reactive healthcare use.^84^, ^95^, ^96^
4. Vaccination. Low GP registration, language barriers, incomplete medical records and limited parental awareness of UK vaccination schedules led to missed vaccinations.^94^, ^95^, ^97^, ^98^

### 3.4 Theme 2: General practice engagement is characterized by a mutual incomprehension and incongruence of UK‐CEE and UK healthcare staff expectations

#### 3.4.1 UK‐CEE community member expectations of general practice

Experiences of state and frequently private healthcare in individuals' home nation shaped healthcare expectations of UK general practice.^16^, ^33^, ^99^ This led to a mutual incompatibility in expectations, with different and thus unfamiliar care arrangements affecting engagement and trust. Examples included:

1. *Cervical smears* undertaken by nursing staff every 3 years.^39^ Individuals frequently underwent annual checks within their home nation for a ‘second opinion’.^39^, ^70^ Limited health literacy and procedural understanding created distrust amongst some, including Roma.^100^
2. *Primary care in pregnancy and post‐partum* undertaken by GPs and ‘non‐doctors’.^39^, ^66^ Health visitors were generally viewed positively, if with confusion,^81^, ^83^ with higher uptake amongst Poles than Slovaks.^67^
3. *Differing child vaccination schedules*, with some vaccination refusal (influenza), missed doses and subsequent infections.^76^, ^97^, ^101^, ^102^
4. *Health prevention*, with low interest and uptake through GP services, for example, smoking, alcohol and dietary advice.^32^, ^68^, ^83^
5. *GP telephone triage and consultations* being viewed negatively due to perceived incomplete assessment, time pressure, language difficulties and lack of physical examination.^32^, ^82^

#### 3.4.2 Appointment booking

GP appointment availability and timeliness were a frequent concern,^39^, ^62^, ^65^, ^66^, ^67^, ^80^, ^99^ particularly for childhood illness.^33^, ^67^, ^81^ Barriers to appointment booking included communicating with reception, inflexible booking mechanisms, refusal of same day appointment requests and interpreter request procedures.^77^ Reception staff were often seen as unsympathetic, condescending and seeking to get rid of patients.^77^, ^84^ Difficulty arranging GP recommended follow‐up appointments, understanding written or telephone communication, rebooking appointments and long waiting times all caused frustration.^77^

Further difficulties included signing in (reception, electronically)^84^ and interpreter absence. Perceived or actual difficulties with appointment access risked individual deregistration^77^ and promoted unscheduled healthcare use, including EDs and walk‐in centres.^31^, ^62^, ^65^, ^81^, ^83^, ^99^, ^103^, ^104^, ^105^

#### 3.4.3 UK‐CEE community members' expectations of general practitioners

Individuals often delayed presentation until they felt that their needs were serious. GP expertize was sought for specific ‘strong’ treatment or specialist referral.^33^ Management decisions using shared decision‐making, nonspecific medication, limited antibiotic prescription, promotion of self‐care and lifestyle advice and ‘watchful waiting’ did not meet these health expectations.^33^, ^65^, ^70^ The short duration of GP or nurse assessment and frequent absence of physical examination were perceived as unthorough and thus incomplete.^32^, ^68^, ^71^, ^77^, ^96^ This mismatch in expectations was compounded by language, cultural and system barriers.

#### 3.4.4 Medication prescription

Antibiotics were felt to be required rapidly for infections, particularly in children. Recommendation of ‘low‐strength’ ‘nonspecific’ over‐the‐counter medications (particularly paracetamol) and antibiotic nonprescription were frequent points of contention.^32^, ^33^, ^39^, ^65^, ^67^, ^71^ Actual or anticipated GP nonprescription led to community discussion of alternative sources, including the ED and (pre‐emptive) purchase from home nation pharmacies or UK‐based Polish supermarkets.^32^, ^33^, ^66^, ^67^, ^81^, ^86^ The threshold for prescribing medications for mental health was seen as too low and failed to address the perceived reactive cause.^71^, ^77^, ^106^

#### 3.4.5 Referral

Prior experience of direct specialist access led to dissatisfaction with the gatekeeper model of care. Some perceived GPs to deliberately obstruct their request for referral.^32^, ^39^, ^70^, ^71^, ^80^, ^84^, ^86^ Self‐care advice and ‘watchful waiting’ provided little relief, led to frustration at a lack of alternative UK healthcare options and indirectly promoted repeated GP or direct hospital attendance to obtain specialist review for themselves or their child.^32^, ^39^ When referred, referral times were seen as too lengthy.^33^, ^62^, ^67^, ^71^, ^77^, ^86^, ^107^, ^108^

#### 3.4.6 Understanding GP care

The reason for perceived denial of care was often not explained to or understood by UK‐CEEs. Some individuals reported being told their requests were not safe, indicated or evidence based.^65^, ^66^, ^67^, ^97^ This failed to address health concerns or validate efforts taken to obtain a GP appointment. Individuals felt that they had not been taken seriously, had been ‘failed’ by their GP and had lost control over their health.^80^ UK‐CEEs desired to know GPs' treatment rationale,^32^ proposing factors including:

1. The system: To limit resource expenditure due to underfunding.^32^, ^66^, ^89^
2. The clinician: Undertraining or incompetence,^65^, ^67^ rude or prejudiced attitude,^39^, ^77^, ^84^ desire to prioritize personal profits^71^ or to end the consultation.^32^, ^77^

Some CEEs felt that their views were confirmed by antibiotic and analgesic prescription or radiological investigation on ED attendance.^62^, ^65^, ^81^, ^83^

#### 3.4.7 Positive aspects of general practice and the NHS

Some positive aspects of general practice and the wider UK health service were reported. These included:

1. *Universal access*, particularly by individuals with low or unstable income and the marginalized, for example, substance misusers.^33^, ^65^, ^109^
2. *Free or lower costs*, associated with a wider range of prescriptions, vaccinations and screening compared with home nations (Poland, Romania, Bulgaria).^33^, ^39^, ^67^, ^68^, ^71^, ^97^
3. *Invitation reminders* for screening.^39^
4. Emergency care services, which were seen as responsive.^16^, ^33^, ^65^, ^67^, ^82^
5. *Healthcare facilities and equipment*, which were viewed as being of a higher standard than within individuals' home nations.^32^, ^67^
6. *Relational care*, including clinician politeness, contrasting them with home nation doctors (Poland). Children felt involved in care decisions.^65^, ^66^, ^67^, ^71^, ^110^
7. *GP health system guidance*, particularly where this allowed efficient navigation of other health services.^33^, ^111^, ^112^
8. *GP promotion of self‐care and avoidance of overtreatment*, a view associated with other broader markers of acculturation into UK society.^32^, ^71^

#### 3.4.8 General practice staff perceptions of UK‐CEE community members

General practice clinicians' and administrators' perceptions of UK‐CEE community members varied from feeling that health needs were well met,^96^ to having differing or unrealistic health expectations^67^ and service misuse.^81^, ^103^ Recognized service barriers included language barriers and interpreter availability,^77^, ^81^, ^96^ limited continuity of care and medical records^75^, ^96^ and variable knowledge of community members' identity, values and culture.^77^, ^96^, ^113^ Some clinicians felt that UK‐CEE individuals needed to change to improve engagement.^32^, ^77^ Others took proactive approaches, including culturally adapted clinics,^35^, ^77^, ^109^ translation services,^84^ resources in different languages,^62^ staff cultural competency training, community staff members and development workers^35^, ^81^, ^84^ and amended consultation formats.^71^, ^81^, ^103^

### 3.5 Theme 3: Perceived or actual unmet expectations embed community distrust and dissatisfaction with general practice

#### 3.5.1 A journey of distrust and dissatisfaction with general practice

Dissatisfaction with UK General Practice was commonplace, shaped by misinformation and negative stereotypes. Limited health system understanding and entitlements predisposed individuals to difficulty overcoming structural barriers to care access, unmet health expectations and negative GP experiences.^31^, ^39^, ^67^, ^80^, ^83^, ^85^, ^86^ Perceived denial of treatment at times of need (e.g., unwell child,^32^, ^33^, ^62^, ^65^, ^67^, ^86^ pregnancy^66^ and precancerous changes on screening)^39^ led to individuals choosing trusted culturally and linguistically familiar care.^33^ Distrust of General Practice and wider government structures^83^, ^100^, ^102^, ^107^ was propagated by co‐nationals and mother tongue media, irrespective of healthcare engagement, with positive NHS experiences attributed to ‘luck’.^33^, ^65^, ^67^, ^97^

#### 3.5.2 Taking back control

Strategies to overcome the perceived power imbalance in accessing GP care included the following:

1. *Culturally familiar healthcare facilities* within the United Kingdom (Polish clinics and pharmacies) and home nations (physical, telephone and video consultations).
2. *Self‐sourcing of treatments* including purchases within home nations or UK Polish supermarkets.
3. *Persistence and pressure on GPs*, through repeated attendance and treatment or referral insistence.
4. *Requesting and collecting health documentation*, particularly for Roma individuals.
5. *Community support network and online forum use* for health and self‐care advice.

#### 3.5.3 Culturally familiar healthcare

Widespread transnational healthcare use was seen as a perceived alternative to GP engagement.^16^, ^33^, ^39^, ^65^, ^66^, ^67^ Liberal prescribing and investigation practices within private or transnational healthcare conflicted with more restrictive evidence‐based practices within the United Kingdom.^33^, ^67^ Faced with seemingly incompatible health offerings, individuals often chose the system they trusted and felt best met their needs.^33^, ^39^, ^67^ Transnational healthcare use was influenced by lack of GP registration, appointment convenience and timeliness^39^; a desire for language and cultural familiarity,^39^, ^65^, ^66^ trusted opinion or specialist review;^67^ and maintenance of personal and healthcare connections in case of future return migration.^39^, ^66^, ^67^ Trips were seen as cost‐effective, particularly if recovery (and thus nonemployment) was required.^33^, ^86^ Cost and limited social networks were barriers to transnational or private healthcare, particularly in the case of insecure employment or residency status.^33^, ^108^ Lack of consistent UK general practice use affected community members' service familiarity and continuity.^27^, ^33^, ^39^, ^80^

#### 3.5.4 Conflicting medical advice and acculturation

At a deeper level, the choice between UK and transnational or private healthcare was an outworking of perceived and desired national identity.^33^ Prioritisation of UK general practice was often associated with a desire to integrate into UK society. Such individuals tended to be younger, (post)graduates, fluent in English and intend to remain in the United Kingdom.^39^, ^64^, ^71^ Dissipation of GP service distrust was gradual (if at all), influenced by peer perceptions, cyclical and potential return migration.^33^, ^62^, ^77^, ^114^ Nonprioritization of health, accumulated stress from nonacculturation, loss of home nation connections and insurance coverage led some to experience a health crisis.^27^ Lack of familiarity with general practice complicated access at that point, negatively impacting health outcomes.^27^, ^33^

### 3.6 Longitudinal consideration

An initial emphasis on immigration‐related service pressures^31^, ^79^ transitioned to more specific aspects of general practice access and engagement. Despite long‐term intentions to remain in the United Kingdom,^62^ nonacculturation to UK health norms, GP services and healthcare entitlements often persisted over time.^33^, ^39^, ^67^, ^77^, ^108^

The Brexit referendum created heightened feelings of instability, future uncertainty, being ‘unwelcomed’ by UK nationals and distrust towards UK authorities.^62^, ^69^, ^77^, ^88^, ^107^, ^115^ Some researchers feared that discussing Brexit would risk community nonengagement or misinterpretation.^77^, ^88^ Indirect implications of Brexit for UK‐CEEs included (1) uncertainty regarding health and social entitlements^77^; (2) deteriorating mental health and wellbeing (mood, anxiety and stress)^107^; and (3) increased perceived or actual discrimination.^62^ This was pronounced for UK‐CEE Roma, with financial benefit ineligibility due to ‘immigration status’.^77^, ^95^ The direct effect of COVID‐19 on UK‐CEE GP perceptions and engagement was not described.

## 4 DISCUSSION

### 4.1 Summary of key findings

This is the first review to focus on CEEs' engagement with and perceptions of UK general practice. Systematic scoping of academic and grey literature allowed consideration of heterogeneous community subgroups. The review identifies general practice under‐registration. Service use is associated with UK‐CEEs' perceived ability, desire and need to engage with general practice. Engagement is characterized by UK‐CEE patients and GP staff failing to comprehend each other's expectations of care. UK‐CEEs' perceived or actual unmet expectations reinforce feelings of distrust and dissatisfaction with general practice, promoting alternative health‐seeking behaviours.

### 4.2 General practice usage and engagement by the UK‐CEE community

#### 4.2.1 Barriers to GP registration and engagement

Low levels of GP registration and barriers to service engagement have been reported within other UK migrant and marginalized groups.^116^, ^117^, ^118^, ^119^ While some barriers such as inadequate documentation are shared with these groups,^120^ the current review finding of a lack of desire to register or engage with GP services seems more specific to the UK‐CEEs. Unofficial employment and accommodation increases the likelihood of registration rejection for UK‐CEE nationals, including homeless, trafficked or Roma individuals.^33^, ^73^, ^74^, ^76^, ^77^ The absence of residency means that individuals' concerns about healthcare charges or deportation are not unfounded.^76^, ^77^

Rather than being a ‘hard to reach community’,^67^, ^77^, ^84^ UK‐CEE individuals may be victims of ‘hard to access’ GP services.^121^ Provision of accessible health information on arrival to a country positively influences engagement with general practice.^122^ Variable accessibility, knowledge and understanding of information on the UK health system (including general practice) impacted UK‐CEEs' ability to access and make decisions around care.^33^, ^67^, ^68^, ^70^, ^72^, ^80^ Disinformation from peers and online forums led to non‐ or limited engagement, perpetuating unfamiliarity with UK general practice and delaying or preventing a convergence of health expectations.^33^, ^65^ Cultural adaptation of care can overcome barriers to general practice engagement, improving responsiveness to individual needs, including disease prevention, within the heterogeneous UK‐CEE community.^77^, ^79^, ^81^, ^83^, ^120^, ^123^

Close working with UK‐CEE community organisations and decision‐makers presents an opportunity to improve care access and empowerment, overcome preconceptions and support alternative approaches to health service provision.^88^, ^124^, ^125^, ^126^ Health outreach for UK‐CEEs through workplaces and homeless services has been shown to be successful.^74^, ^80^, ^127^ A codesigned reconceptualization of GP services incorporating proactive outreach alongside ‘out of hours’ style drop‐in options would reduce barriers to engagement. Such changes would require sustained political will and investment.

#### 4.2.2 Continuity of care

The current review frequently identified a UK‐CEE reliance on transnational or emergency care. Similar health practices have been described for CEE migrants in other European countries.^67^, ^86^, ^128^, ^129^, ^130^ Transnational and private healthcare usage can be a strategy to regain equality and control over health and healthcare expectations.^35^, ^131^, ^132^ Disjointed care risks missed or late diagnoses, compromised infectious disease prevention and child health surveillance.^68^, ^74^, ^95^, ^133^ The current review suggests that transiency and cyclical migration may exacerbate such concerns in the UK‐CEE community.^74^, ^134^ UK‐CEE health knowledge and responsibility in health decision‐making have been suggested to influence the perceived value of health prevention and care continuity.^135^

#### 4.2.3 Overcoming community member dissatisfaction and distrust

Review findings suggest that UK‐CEE patients and GP staff often fail to comprehend each other's expectations of care. The resultant negative perceptions of general practice affect the degree of service engagement (Figure 2). UK‐CEEs' direct comparison of general practice with private consumer‐focussed ‘culturally familiar’ healthcare reinforces a belief of GP inaccessibility and unresponsiveness.^33^, ^71^, ^77^, ^88^ Experiential knowledge, perceived need, accessibility and practitioner expertize all influence community members' sense of ‘candidacy’ towards UK healthcare.^33^, ^136^, ^137^ Experiential knowledge is recursively shaped by positive experiences of home nation healthcare and negative experiences of UK general practice, creating a framework for understanding decision‐making around ongoing general practice engagement.^137^ Positive experiences of general practice are required to change care‐seeking behaviours and recursively shape UK‐CEEs' judgements of candidacy, accessibility and expertize.^137^

UK‐CEE community members' desire to avoid healthcare disempowerment, inequity and perceived or actual prejudice is understandable.^32^, ^33^, ^138^ Coproduced service reconceptualization alongside community outreach increases awareness, trust and accessibility.^139^, ^140^ The breadth of publications within the current review suggests a willingness to shape care design and delivery through interviews and focus groups. Strategies utilized for UK‐CEE recruitment and engagement warrant greater exploration to determine ‘best practice’.

#### 4.2.4 Overcoming structural and clinical barriers

We identified culturally tailored approaches to overcome barriers to care access, including different language resources on service use, registration support, community staff and mediators, and collaboration with community organisations.^35^, ^62^, ^77^, ^84^ Culturally sensitive responses to concerns on appointment availability, health prevention and screening, and consultation, prescribing and referral approaches would improve perceptions of general practice.^141^ This may necessitate critical appraisal of the current policy‐driven approach to GP care within the United Kingdom, particularly in terms of its definition and implicit discouragement of perceived health service ‘over‐utilization’. This view is supported by the impact of national policies, including the ‘NHS Visitor and Migrant Cost Recovery Programme Implementation Plan’ on delayed treatment for migrants (particularly undocumented migrants) and wider public health.^142^

To improve CEE satisfaction with and quality of GP care, service reappraisal will need to consider service provider and UK‐CEEs' expectations, preconceptions and prejudices.^133^, ^143^, ^144^ Our findings suggest that general practice staff members' attitudes and understanding of UK‐CEE health beliefs, expectations and dissatisfaction vary.^27^, ^35^, ^68^, ^77^, ^81^, ^84^, ^92^ This is in keeping with previous reports advocating for confrontation^145^ or abdication of meeting UK‐CEE health expectations.^67^, ^77^ An inability to meet differing health expectations indicates a limitation of GP and NHS procedural policy and is, in effect, making a judgement as to who is a suitable candidate for care. This is demonstrated by resultant UK‐CEE ‘service misuse’, perceived barriers and nonengagement,^71^ unmet health needs, negative service perceptions and alternative healthcare‐seeking behaviours.^121^, ^144^, ^146^ The current review identified a need for greater UK GP awareness of international health systems and cross‐cultural communication skills training. Normalisation process theory and participatory learning and action (PLA) have previously been used to collaboratively adapt and implement migrant care training and guidance within general practice.^147^ Such approaches offer the potential to develop mutually agreeable GP care strategies tailored to UK‐CEE patients.

Brexit unsettled many UK‐CEE community members, challenging feelings of belonging, entitlement and trust.^24^, ^62^, ^69^, ^77^, ^107^, ^115^, ^148^ Difficulties and concerns in applying for or being granted (pre‐)settled status, particularly for vulnerable CEE nationals, have been reported,^149^, ^150^ alongside presumed loss of access to general practice services,^77^, ^151^ payment requirements or disclosure to UK authorities.^33^, ^81^ In keeping with this,^152^ describe the impact of dynamic power structures within NHS maternity services on care for undocumented migrants. The associated tensions between healthcare and immigration systems create barriers to access for migrants and conflict with principles of patient‐centred care.^152^

COVID‐19‐related restrictions, safety concerns and remote consultation will influence UK‐CEEs' engagement with GP services and transnational healthcare.^32^, ^82^, ^92^, ^153^, ^154^, ^155^, ^156^ Recording UK‐CEEs as ‘White’ or ‘White Other’ in population^63^, ^73^, ^74^, ^127^ and COVID‐19 data^157^ prevents disaggregation by ethnicity. Longitudinal monitoring of UK‐CEE general practice engagement is required to measure the impact of Brexit and COVID‐19 on community health equity^27^, ^69^, ^81^, ^158^ and inform service investment.^16^, ^68^, ^69^, ^159^ Increased UK‐CEE general practice registration would provide a semi‐comprehensive health information source enabling this.^27^, ^160^, ^161^, ^162^

### 4.3 Implications for future practice, policy and research

Overcoming UK‐CEE dissatisfaction with general practice is required to increase registration rates and service knowledge.^121^ UK‐CEE involvement in shaping service delivery and communication skills training would enable empowerment through cultural adaptation of services. Effective strategies for targeting specific UK‐CEE community health needs, including disease prevention, child and maternal health, and mental health, would offer significant health benefits.^27^, ^74^ The absence of cohesive local and national data to monitor UK‐CEE community health needs and service engagement requires urgent policymaker consideration.^63^, ^73^, ^74^, ^127^ Consideration of the impact of Brexit on the health of CEE communities, particularly for smaller national and marginalized subgroups, is required in both the acute and long term.

### 4.4 Strengths and limitations of the review

The review provides a comprehensive exploration of academic and grey literature,^40^, ^44^, ^57^ demonstrating a model for identifying service development requirements,^163^ future research and intervention development.^164^ Critical appraisal aids interpretation of findings,^40^ informing subsequent research into the effect and transferability of outcomes.^35^, ^80^ Findings were validated by CEE community members (Romanian, Polish and Lithuanian). We recognize that certain subgroups (e.g., trafficked individuals),^73^ stigmatized conditions (e.g., mental health, alcohol or substance misuse)^27^ and devolved nations (Northern Ireland, Wales) may be underrepresented. The recency of Brexit and the COVID‐19 pandemic resulted in limited data for consideration. Both events will influence the implementation of findings at a local and national level.

## 5 CONCLUSION

A large and increasingly established community of CEE nationals live and work in the United Kingdom. Their ability, desire and need to register and engage with general practice services are shaped by an intersection of individual cultural and sociodemographic factors. Difficulties overcoming structural and in‐consultation barriers to care are common, with health expectations often going unmet. Negative experiences heighten pre‐existing mistrust and dissatisfaction with UK General Practice, while promoting alternative help‐seeking strategies such as self‐care, ED use and private ethnic and transnational healthcare. Marginalized UK‐CEE community subgroups have particularly poor general practice service engagement and outcomes, including Roma, trafficked and homeless individuals. Further primary research is required to identify what is likely to work for which CEEs and in which context. Overcoming barriers to care requires trust between community members and general practice services. Community codesign approaches may support service access, information provision, communication and health management for CEEs. The current review is particularly timely, given the influence of Brexit and the COVID‐19 pandemic on migrant health and health service engagement.

## AUTHOR CONTRIBUTIONS

Aaron Poppleton, the lead researcher, designed the review protocol and implemented the database searches, data extraction and coding and is also the primary review author. Kelly Howells was involved in title and abstract screening, development of data abstraction template, data extraction, coding and cross‐checking and provided review and feedback on the manuscript. Isabel Adeyemi was involved in title and abstract screening, development of data abstraction template, data extraction, coding and cross‐checking and provided review and feedback on the manuscript. Carolyn Chew‐Graham was responsible for academic supervision of the primary review author, including discussion and formulation of thematic concepts and ideas presented within the discussion, and provided review and feedback on the manuscript. Lisa Dikomitis was responsible for academic supervision of the primary review author, including discussion and formulation of thematic concepts and ideas presented within the discussion, and provided review and feedback on the manuscript. Caroline Sanders was responsible for academic supervision of the primary review author, discussion and feedback of the review protocol, including conceptual and methodological development, oversight of any disagreements relating to publication screening and inclusion and discussion and formulation of thematic concepts and ideas presented within the discussion and also provided review and feedback on the manuscript.

## CONFLICT OF INTERESTS

The authors have no conflicts of interest to declare. All co‐authors have seen and agree with the contents of the manuscript and there is no financial interest to report. We certify that the submission is original work and is not under review at any other publication.

## Supporting information

Supporting information.

Supporting information.

Supporting information.

Supporting information.

## Data Availability

Data sharing is not applicable to this study as no new data were created or analysed.
