# Supplementary material for: The perceptions of general practice among Central and Eastern Europeans in the United Kingdom: A systematic scoping review
Source: Health Expect. 2022 Jan 19;25(5):2107–23. doi: 10.1111/hex.13433 (PMC9615075; doi:10.1111/hex.13433)
Supplement: Supplementary file 1 — Supporting information. [file HEX-25--s003.docx]

## **Appendix A. Publication database search terms and structure**

### **Core database search structure with terms listed by concept category**

| **Concept 1: Central and Eastern European** | Polish or Poland or pole* or Romania* or Hungar* or Bulgar* or Lithuan* or Latvia* or Latvian* or Croatia* or Czech* or Estonia* or Slova* or Sloveni* or EU or European Union or Eastern Europe* or Central Europe* |
| --- | --- |
| **Concept 2: Migration** | migrant* or migration or immigrant* or immigration or traffic* |
| **Concept 3:** **United Kingdom** | UK or United Kingdom or Britain or England or Scotland or Wales or Northern Ireland or London |
| **Concept 4: General Practice** | general practi* or family practi* or family physician* or primary health* or primary care or family doctor* or ambulatory care or GP |

### Adapted database searches

### **Scopus search:**

TITLE-ABS-KEY(Polish or Poland or pole* or Romania* or Hungar* or Bulgar* or Lithuan* or Latvia* or Latvian* or Croatia* or Czech* or Estonia* or Slova* or Sloveni* or EU or "European Union" or "Eastern Europe*" or "Central Europe*") And TITLE-ABS-KEY(migrant* or migration or immigrant* or immigration or traffic*) And TITLE-ABS-KEY(UK or "United Kingdom" or Britain or England or Scotland or Wales or "Northern Ireland" or London) And TITLE-ABS-KEY("general practi*" OR "family practi*" OR "family physician*" OR "primary health*" OR "primary care" OR "family doctor*" OR "ambulatory care" OR "emergency department" OR "emergency service*" OR "accident and emergency" OR "acute care" OR "NHS" OR "National Health Service" OR "Walk in centre" OR GP) AND PUBYEAR > 2003 AND ( LIMIT-TO ( AFFILCOUNTRY,"United Kingdom" ) ) AND ( LIMIT-TO ( LANGUAGE,"English" ) )

### **IBSS Search:**

(Polish OR Poland OR pole* OR Romania* OR Hungar* OR Bulgar* OR Lithuan* OR Latvia* OR Latvian* OR Croatia* OR Czech* OR Estonia* OR Slova* OR Sloveni* OR EU OR "European Union" OR "Eastern Europe*" OR "Central Europe*") AND (migrant* OR migration OR immigrant* OR immigration OR traffic*) AND (UK OR "United Kingdom" OR Britain OR England OR Scotland OR Wales OR "Northern Ireland" OR London) AND ("general practi*" OR "family practi*" OR "family physician*" OR "primary health*" OR "primary care" OR "family doctor*" OR "ambulatory care" OR "emergency department" OR "emergency service*" OR "accident and emergency" OR "acute care" OR "NHS" OR "National Health Service" OR "Walk in centre" OR GP)

### **Sociological Abstracts Search:**

(Polish OR Poland OR pole* OR Romania* OR Hungar* OR Bulgar* OR Lithuan* OR Latvia* OR Latvian* OR Croatia* OR Czech* OR Estonia* OR Slova* OR Sloveni* OR EU OR "European Union" OR "Eastern Europe*" OR "Central Europe*") AND (migrant* OR migration OR immigrant* OR immigration OR traffic*) AND (UK OR "United Kingdom" OR Britain OR England OR Scotland OR Wales OR "Northern Ireland" OR London) AND ("general practi*" OR "family practi*" OR "family physician*" OR "primary health*" OR "primary care" OR "family doctor*" OR "ambulatory care" OR "emergency department" OR "emergency service*" OR "accident and emergency" OR "acute care" OR "NHS" OR "National Health Service" OR "Walk in centre" OR GP)
