# Supplementary material for: The perceptions of general practice among Central and Eastern Europeans in the United Kingdom: A systematic scoping review
Source: Health Expect. 2022 Jan 19;25(5):2107–23. doi: 10.1111/hex.13433 (PMC9615075; doi:10.1111/hex.13433)
Supplement: Supplementary file 2 — Supporting information. [file HEX-25--s002.docx]

## **Appendix B. Grey literature databases and search terms**

|  | ***Source*** |
| --- | --- |
| ***Online databases*** | *· Electronic Thesis Online Service (ETHOS - British Library)*  *· Web of Science: Conference Proceedings Citation Index*  *· Open Grey* |
|  | *Search terms*  *· Polish OR Poland OR Pole OR Romania OR Romanian OR Hungary Or Hungarian OR Bulgaria OR Bulgarian OR Lithuania OR Lithuanian OR Latvia OR Latvian OR Croatia OR Croatian OR Czech OR Estonia OR Estonian OR Slovak OR Slovakia Or Slovakian OR Slovenia Or Slovenian OR Eastern Europe OR Eastern European OR Central Europe OR Central European*  *AND*  *· GP OR General Practice OR General Practitioner OR Primary Care* |
| ***Online repositories*** | *·* [*Healthcare Improvement Scotland.*](http://www.healthcareimprovementscotland.org/about_us.aspx) *Published Resources.* [*http://www.healthcareimprovementscotland.org*](http://www.healthcareimprovementscotland.org)  *·* [*National Institute for Health and Care Excellence*](http://www.nice.org.uk/aboutnice/) *(NICE). NHS National Institute for Health and Care Excellence*  *·* [*http://www.nice.org.uk/*](http://www.nice.org.uk/)  *·* [*National Institute for Health Research*](http://www.io.nihr.ac.uk/what-we-do/)*. (NIHR). Innovation Observatory*  *·* [*http://www.io.nihr.ac.uk/*](http://www.io.nihr.ac.uk/)  *·* [*UK Department of Health*](http://www.nric.org.uk/about) *(NHS). International Resource for Infection Control (iNRIC)*  *·* [*http://www.nric.org.uk/resources*](http://www.nric.org.uk/resources)  *·* [*National Health Service UK*](https://www.england.nhs.uk/about/) *(NHS). NHS England*  *·* [*http://www.england.nhs.uk/*](http://www.england.nhs.uk/) |
|  | *Search terms*  *· “Central European” “GP”*  *· “Eastern European” “GP”*  *· “Polish” “GP”*  *· “Poland” “GP”*  *· “Romania” “GP”*  *· “Romanian” “GP”*  *· “Central European” “General Practice”*  *· “Eastern European” “General Practice”*  *· “Polish” “General Practice”*  *· “Poland” “General Practice”*  *· “Romania” “General Practice”*  *· “Romanian” “General Practice”* |
| ***Library catalogue*** | *OpenDOAR: Directory of Open Access Repositories*  [*The University of Manchester - Institutional Repository*](http://v2.sherpa.ac.uk/id/repository/3818?template=opendoar)  *The University of Glasgow - Enlighten*  *University College London - UCL Discovery* |
|  | *Search terms*  *· “Central European” “GP”*  *· “Eastern European” “GP”*  *· “Polish” “GP”*  *· “Poland” “GP”*  *· “Romania” “GP”*  *· “Romanian” “GP”*  *· “Central European” “General Practice”*  *· “Eastern European” “General Practice”*  *· “Polish” “General Practice”*  *· “Poland” “General Practice”*  *· “Romania” “General Practice”*  *· “Romanian” “General Practice”* |
| ***Online search engines*** | *Google (*[*www.google.co.uk*](http://www.google.co.uk)*)*  *Google Scholar (*[*http://scholar.google.com*](http://scholar.google.com)*)* |
|  | *Search terms*   - "Eastern European" "General Practice" "migrant" - "Central European" "General Practice" "migrant" |
