# Supplementary material for: The perceptions of general practice among Central and Eastern Europeans in the United Kingdom: A systematic scoping review
Source: Health Expect. 2022 Jan 19;25(5):2107–23. doi: 10.1111/hex.13433 (PMC9615075; doi:10.1111/hex.13433)
Supplement: Supplementary file 3 — Supporting information. [file HEX-25--s001.docx]

## **Appendix C. List of studies included within the review**

| **Study Authors, year, title** | **Publication type** | **Study type** | **Location or setting of study** | **Sample size & population(s) (including nationalities)** | **Year of data collection** |
| --- | --- | --- | --- | --- | --- |
| Bell, S., et al. (2019). "'I don't think anybody explained to me how it works': qualitative study exploring vaccination and primary health service access and uptake amongst Polish and Romanian communities in England." | Research paper | Qualitative (Interviews) | England in 3 geographical areas: Boston, Lincolnshire; Slough, Berkshire; Brent, London. However due to recruitment strategy (social media) they did recruit some participants out of area. | 20 Polish and 10 Romanians in the community were interviewed. 20 healthcare workers were also interviewed. Nationalities of healthcare workers is not listed, but the results indicate they were not Polish or Romanian due to communication issues. | 2018 |
| Bell, S., et al. (2020). "What have we learnt from measles outbreaks in 3 English cities? A qualitative exploration of factors influencing vaccination uptake in Romanian and Roma Romanian communities." | Research paper | Qualitative (Interviews) | Study took place in Birmingham, Leeds and Liverpool as local measle outbreaks in these communities particularly affected Romanian and Roma Romanian communities. | 33 providers (which included frontline vaccination workers) and 9 community members. The community members were all female: 6 Romanian and 3 Roma Romanian. | 2017 to 2018 |
| Bielecki, K., et al. (2019). "Low uptake of nasal influenza vaccine in Polish and other ethnic minority children in Edinburgh, Scotland." | Research paper | Quantitative (Retrospective cohort study) | 3 primary schools in Edinburgh, Scotland | 922 pupils in the cohort: 387 Polish; 297 White British; 171 Other identified ethnic minority. | 2016 to 2017 |
| Blake, H., et al. (2018). "Employee perceptions of a workplace HIV testing intervention." | Research paper | Mixed methods (survey, interviews) | Organisations in a target area of England were invited to participate in the study but the location of these organisations are not discussed in this paper. | 11 organisations agreed to take part in the study, which involved health checks at a 'healthy hub roadshow.' 776 employees attended the roadshow and 771 completed the exit questionnaire 51.4% male; 4.7% Sub-Saharan Africa; 15.75% Eastern European; 1.8% Western European; 1% Caribbean; 6.3% Asian. 35 interviews were completed with male migrant workers (Sub-Saharan Africa n=10; Eastern European n= 12; Asian n=11 Western European and Caribbean =2). | 2016 |
| Bosqui, T., et al. (2019). "First-generation migrants' use of psychotropic medication in Northern Ireland: a record linkage study." | Research paper | Quantitative - Other (a record linkage study) | Northern Ireland | 1,019,759 people were included; 49,342 born outside of the UK and ROI. The biggest migrant group was Polish (n= 14,995) followed by Lithuania (n=5478) and India (n=3899) | 2011 |
| Brawley, D., et al. (2013). "The care of trafficked women in an integrated sexual health service: A six-year case-note review." | Oral presentation | Retrospective case series | Case note reviews from sex-trafficked women presenting at sexual health services. Location not specified | Thirty-eight women were identified with a mean age of 24 years (15–41). Sixty-eight percent were from Africa with 32% from Eastern Europe, Asia and South America | 2013 to 2017 |
| Bray, J. K., et al. (2010). "Obstetric care of New European migrants in Scotland: An audit of antenatal care, obstetric outcomes and communication." | Research paper | Audit (Retrospective case audit) | Lothian, Scotland (maternity units) | 114 A8 nationals - Polish 91 (82%) other nationalities not specified beyond A8 | 2006 |
| Burns, F. M., et al., (2011). "Sexual and HIV risk behaviour in Central and Eastern European migrants in London" | Research paper | Quantitative (Survey) | The community sample was recruited in London (Newham and Hammersmith & Fulham) and the web survey was advertised on websites for CEE nationals in London and the UK but Web survey respondents who gave their home post-code outside London were excluded from the study. | 2648 CEE migrants completed the survey. | July 2008 to March 2009 |
| Chojnacki, S. (2020) Psychological Wellbeing of Polish Migrants: What is the Role of the NHS? | Thesis | Mixed Methods (Survey) | Online (UK) | Polish adults living in the UK - 605. 46 NHS Mental Health trusts responded to freedom of information requests | June 2019 to February 2020 |
| Cleland, J. A., et al. (2012). "Community pharmacists' perceptions of barriers to communication with migrants." | Research paper | Qualitative (Interviews) | North East Scotland | 14 community pharmacists took part, 10 of which were women | 2008 |
| Close, C. M., et al. (2018). "Migrant mental health and representation in routine administrative registers." | Research paper | Qualitative (Focus groups) | Northern Ireland | 17 professionals; 15 female, 2 male. All participants either directly supported migrants or worked for an organisation that supports migrants. 6 participants are listed as not being born in the UK or Ireland but their ethnic group is not listed. | 2015 |
| Collinson, S. and R. Ward (2010). "A nurse-led response to unmet needs of homeless migrants in inner London." | Research paper | Quantitative (Survey) | Hackney, London at a mobile run clinic | Compiled a sample of 98 Eastern Europeans and 62 were screened for TB. At additional 15 people came forward for screening not included in the original sample who were not migrants but vulnerable (e.g. homeless). | 2008-2009. |
| Collis, A., et al. (2010). Workers on the move 3: European migrant workers and health in the UK: the Evidence, Keystone Development Trust. | Report | Quantitative (Survey) | Migrant workers living in Thetford and surrounding area who accessed Keystone’s META service | Figure note stated. Participants were Polish, Lithuanian, Latvian and Slovakian. | January and March 2010. |
| Condon, L. J. and D. Salmon (2015). "'You likes your way, we got our own way': Gypsies and Travellers' views on infant feeding and health professional support." | Research paper | Qualitative (Interviews) | City in south west England | 22 mothers and grandmothers of English Gypsy, Irish Traveller and Romanian Roma ethnicity | November 2011 and February 2012 |
| Condon, L. J. and S. McClean (2017). "Maintaining pre-school children's health and wellbeing in the UK: a qualitative study of the views of migrant parents." | Research paper | Qualitative (Focus groups) | U.K. | Romanian = 7 (5 female). Roma = 6 (4 female). Polish = 6 (4 female). Somali = 5 (all female). Pakistani n=4 (all female). | January to March 2010. |
| Cook, J., et al. (2012). "Accession 8 Migration and the Proactive and Defensive Engagement of Social Citizenship." | Research paper | Mixed methods (Focus groups, interviews) | Northern English City. | Purposive sampling used to recruit 89 respondents. Participants were A8 migrants from Polish and Slovak communities | Unclear - likely 2008 |
| Crowther, S. and A. Lau (2019). "Migrant Polish women overcoming communication challenges in Scottish maternity services: A qualitative descriptive study." | Research paper | Qualitative (Interviews) | Urban city in Scotland | 9 in-depth interviews were conducted with Polish women who had given birth within the last 12 months of the study start date. | 2017 to 2018 |
| Evans, A. R., et al. (2009). "The sexual attitudes and lifestyles of London's Eastern Europeans (SALLEE Project): design and methods." | Research paper | Mixed methods (quant: a cross-sectional survey, qual: semi-structured in-depth interviews). | Two London boroughs (Newham and Hammersmith & Fulham). The internet sample was limited to people with a London postcode. | 3,005 CEE migrants (Czech Republic, Estonia, Hungary, Latvia, Lithuania, Poland, Slovakia, Slovenia, and Romania and Bulgaria.) 2,276 respondents recruited in the community sample (response rate of 33.6%), 357 in the clinic sample (response rate of 92.2%) and 372 in the Internet sample. response rate of 92.2%. 40 in-depth qualitative interviews were undertaken with a range of individuals, as determined by the interview quota matrix | 2009 |
| Evans, A. R., et al. (2011). "Central and East European migrant men who have sex with men: An exploration of sexual risk in the UK." | Research paper | Quantitative (Survey) | UK | 691 Central and Eastern European men. Individual country of origin not listed. | 2009 |
| Evans, A., et al. (2010). "Use of sexual and reproductive health services by Central and Eastern European women in London." | Abstract | Quantitative (Survey) | London, UK | 1173 women from 10 CEE countries | 2008 to 2009 |
| Fitzgerald, I. and R. Smoczynski (2017). "Central and Eastern European Accession: Changing Perspectives on Migrant Workers." | report (article providing a reflection on a topic, not peer reviewed) | Other - Policy document. I'm not entirely sure | Not Applicable | Not Applicable | Not Applicable |
| Ford, A., et al. (2013). Cutting A&E use and health inequalities. | Online magazine article | Other - unclear | Merton, south west London | People from eastern Europe and South East Asia | The programme began in 2010. No reported formal data collection strategy. |
| Gill, G. (2009). "The health needs of the Slovak Roma community in Sheffield." | online magazine for community practitioners | Other - report based on case studies | Sheffield | Slovak Roma | "since 2005" |
| Giuntella, O., et al. (2018). "The effects of immigration on NHS waiting times." | Research paper | Other - health economics | England (national) | A8 immigrants | Administrative records data from 2003 to 2012 |
| Gondek, D. and J. B. Kirkbride (2018). "Predictors of mental health help-seeking among polish people living the United Kingdom." | Research paper | Quantitative - survey | The survey was done remotely. Most participants lived in England (74.6%) or Scotland (21.5%), fewer from elsewhere in the UK: 2.8% from wales, 0.7% from northern Ireland, 0.9% missing data. | 536 Polish migrants | August to November 2015 |
| Goodwin, R., et al. (2013). "Perceived Changes in Health and Interactions With "the Paracetamol Force": A Multimethod Study." | Research paper | Mixed methods (sequential study - questionnaires and interviews) | the "sample was from a wide diversity of locations across Britain" | Polish migrants. 418 questionnaire respondents in questionnaire T1, 228 at T2, and 214 at T3. Quantitative data on the 172 participants who took part in all three data waves. In-depth interviews with 30 respondents | June 2007 to February 2009 |
| Gorman, D. and L. Porteous (2018). "Influences on Polish migrants' breast screening uptake in Lothian, Scotland." | Research paper | Qualitative service evaluation | Lothian, Scotland | 11 Polish women | November 2014 to February 2015 |
| Gorman, D. R., et al. (2019). "A qualitative study of vaccination behaviour amongst female Polish migrants in Edinburgh, Scotland." | Research paper | Service evaluation (qualitative) | Edinburgh, Scotland | 13 Polish women | 2018 |
| Gorman, D. R., et al. (2020). "Comparing vaccination hesitancy in Polish migrant parents who accept or refuse nasal flu vaccination for their children." | Research paper | Service evaluation (questionnaires) | Edinburgh, Scotland | 128 Polish parents (37.3% response rate) | 2018 |
| Gorman, D., et al. (2018). "A Review of Suicides of Polish People Living in Scotland." Glasgow: ScotPHN. | Report | Retrospective case series | Scotland | 68 Polish individuals | 2012 to 2016 |
| Hodgekiss, C.H.E. and Shipman, A.R., (2010). Vaccination programme in Poland: are we missing a vulnerable population? | Research paper | Case report | Thames Valley Region, England | Polish | Not stated. Presumed 2009 |
| Hodson, N. and R. Glennerster (2020). "Discharge policies for homeless people and immigrants: Compromising professional ethics." | Research paper | Case study | UK. It is unclear which part of the UK | Lithuanian man | Not reported |
| Ignaszak-Szczepaniak, M., et al. (2009). "Reasons for visiting Polish primary care practices by patients aged 18-44 years: the largest emigrating age group." | Research paper | cohort study (not reported by authors) | Chodziez town, Western Poland, and the surrounding community | Data of GP visits for 12 535 registered Polish patients | June 2005 to May 2006 |
| Jackowska, M., et al. (2012). "Cervical screening among migrant women: a qualitative study of Polish, Slovak and Romanian women in London, UK." | Research paper | Mixed methods (Interviews and focus groups) | London | Study 1 - Professionals: NHS and other professionals with knowledge of cervical screening among CEE migrants (n=11) working with Central and Eastern European migrants Study 2 - 3 Polish, 1 Slovak and 1 Romanian  Study 3 - 11 Polish, 7 Slovak, and 2 Romanian women | 2008–2009 |
| Jackson, C., et al. (2017). "Needles, Jabs and Jags: a qualitative exploration of barriers and facilitators to child and adult immunisation uptake among Gypsies, Travellers and Roma." | Research paper | Qualitative (Interviews) | Six Traveller communities based in the UK (Bristol, Glasgow, York and London). | 174 Travellers, including Roma Romanian, English Gypsy, Slovakian Roma, Glasgow Scottish Show people and Irish Travellers. | December 2013-April 2015 |
| Khalid, T., (2018) Eastern European Research Project | Report | Mixed methods - survey and focus groups | Wakefield, Yorkshire | English and Polish language survey. 157 completed - 81 English 76 Polish. Participants: - Polish (83%), 3% Latvian, 3% Lithuanian, British (5%), individual respondents from Russia, Bulgaria, Romania and the Czech Republic. 6 focus groups in Polish, English and Czech Roma. A total of 56 attended 75% being Polish and the remaining Czech Lithuania, Romania and other national origins. | June to December 2017 |
| Kouvonen et al. (2014) ”We asked for workers, but human beings came” Mental health and well-being of Polish migrants in Northern Ireland | Report | Qualitative | Northern Ireland | Polish | Unclear - prior to 2014 |
| Leaman, A. M., et al. (2006). "Use of the emergency department by Polish migrant workers." | Report | Audit | Telford, UK | 90 patients of Polish origin | 2005 |
| Lehane, D., et al. (2020). "What influences Roma women attending NHS cervical screening? Knowledge, fear, and passive consent." | abstract for oral presentation | Qualitative (interviews) | Sheffield | 16 Slovak Roma women | Not reported |
| Lincolnshire County Council, (2013) Ensuring Inclusive Healthcare in Lincolnshire | Report | Quantitative (Survey) | Lincolnshire | A8 nationals - 131 | Latest 2009 |
| Lindenmeyer A, (2016b). Experiences of primary care professionals providing healthcare to recently arrived migrants: a qualitative study. | Research Paper | Qualitative (Interviews) | Birmingham | Purposive sample of 10 practices. Interviews with 6 general practitioners, 5 nurses and 6 administrative staff. 10 interviewees were from an ethnic minority background; some discussed their own experiences of migration. | Latest 2016 |
| Lindenmeyer, A., et al. (2016a) Health across borders Recent migrants’ experience of managing their health and wellbeing in the UK and the implications for health policy and practice | Report | Qualitative | Birmingham and the Black Country | Polish (4), 12 other non-UKCEE nationalities | Unclear - likely 2014 |
| MacKichan F, et al. (2017) Why do patients seek primary medical care in emergency departments? An ethnographic exploration of access to general practice. | Research paper | Case study (ethnographic) | Three commissioning areas in England | 6 General practices - Participant described as Eastern European | 2012-2015 |
| Madden, H., et al. (2017). ""Always paracetamol, they give them paracetamol for everything": a qualitative study examining Eastern European migrants' experiences of the UK health service." | Research paper | Mixed methods (Qualitative interviews and focus groups) | Warrington (Northwest England) | 42 Eastern European participants. Polish (majority); Belarus, Hungary, Latvia, Russia, Slovakia and Ukraine | June - September 2014 |
| Main, I. (2016). "Biomedical practices from a patient perspective. Experiences of Polish female migrants in Barcelona, Berlin and London." | Research paper | Qualitative - ethnographic | London, Barcelona, Berlin | Polish women, 98 interviews | 2008-2013 |
| Mills, K. and T. Knight (2010). "Offering substance misuse services to Accession Eight migrants in London: Findings from a qualitative study." | Research paper | Qualitative (Interviews) | 7 London boroughs | 20, in-depth interviews Drug and Alcohol Action Teams staff and treatment services, 19 interviews were conducted with related service providers. Six service users | November 2006 and May 2008, |
| O’Neill, B., (2011) Cornwall and Isles of Scilly Primary Care Trust Health Equity Audit 2008/09 | Report | Audit, Case reports | Cornwall | Polish and Lithuanian | 2009 |
| Osipovič, D. (2013). "‘if I get ill, It’s onto the plane, and off to Poland. Use of health care services by polish migrants in London." | Research paper | Qualitative (Interviews) | London | 62 Polish migrants | 2007-2008 |
| Patel, H., et al. (2020). "Awareness of and attitudes towards cervical cancer prevention among migrant Eastern European women in England." | Research paper | Mixed methods (interviews, survey) | University Hospitals Leicester, Northampton General Hospital and University Hospitals of North Midlands, England; Community or in colposcopy clinic | 331 surveys and 46 interviews. Survey - All respondents CEE. Interviews: Polish, Lithuanian, Latvian, Czech, Slovak, Slovenian, Hungarian, Romanian | April 2015 - December 2016. |
| Penn, R., (2008) An assessment of the health needs of eastern European immigrants from the eight accession countries: A report to Central Lancashire Primary Care Trust | Report | Mixed methods - surveys and interviews | Central Lancashire | 102 respondents - Mostly Polish, some English. Respondents (A8): Poland (overwhelmingly). Far fewer reports of clients from Slovenia, Slovakia, Latvia, Lithuania, and the Czech Republic. None from either Hungary or Estonia. | 2008 |
| Phillimore, J. (2011). "Approaches to health provision in the age of super-diversity: accessing the NHS in Britain's most diverse city." | Research paper | Mixed-methods (Interviews, focus groups, survey) | Birmingham - North west of city. areas with high concentrations of new arrivals. Predominantly unemployed, working in low income employment or not permitted to work, | N=189 questionnaires - Poland (25%), eight focus groups Included Polish families and singles. Eight community leaders - one represented the Polish community | 2008 |
| Platt, L., et al. (2011). "Risk of sexually transmitted infections and violence among indoor-working female sex workers in London: the effect of migration from Eastern Europe." | Research paper | Quantitative (Cross-sectional survey) | London | 268 participants, 61% from EE/FSU. 30% from Romania, 19% from Lithuania, 17% from Poland, 7% from Latvia and Albania, 5.5% from Czech Republic, and 4% from the Russian Federation. 2% or less originated from Bulgaria, Slovakia, Kosova, Estonia, Moldova, Serbia or Tajikistan. Median duration in the UK was 3 years (IQR 0.6e10) | September 2008 and July 2009 |
| Pollock., et al. (2007) Q Fever in Migrant Workers, Scotland | Letter | Retrospective cohort study | Stirling, Scotland | British, Slovakia (41), Poland (3), Czech Republic (2), and Lithuania (2) | June 2006 |
| Pope, C., et al. (2019). "Navigating and making sense of urgent and emergency care processes and provision." | Research paper | Qualitative (Citizens panels, interviews) | Geographical area covering four English counties | CEE participants - Polish, ?others - however not stated - 24 people for community groups (12 CEE). 100+41 interviewed individuals | September 2016 to July 2017 |
| Richards, J., et al. (2014). "Maternal and infant health of Eastern Europeans in Bradford, UK: a qualitative study." | Research paper | Qualitative (Interviews) | Bradford, England | Polish + Slovak (Roma). Staff exposed to Eastern European populations (n:11). 2 Voluntary workers, 4 health visitors and 5 community midwives. | Not stated |
| Selkirk, M., et al. (2012). "Influences on Polish migrants' responses to distress and decisions about whether to seek psychological help." | Thesis | Qualitative (Interviews) | Forth Valley, Falkirk, Scotland | 9 Polish participants - 7 Community members and 2 key informants | Unclear (2010 or earlier) |
| Sharp, C. and G. Randhawa (2015). "UK Polish Migrant Attitudes Toward Deceased Organ Donation: Findings from a Pilot Study." | Research paper | Qualitative (Interviews) | Luton & Dunstable, England | 31 Polish migrants | May to November 2011 |
| Sharp, C. and G. Randhawa (2016). "The Potential Role of Social Capital in the Willingness to be a Deceased Organ Donor: A Case Study of UK Polish Migrants." | Research paper | Qualitative (Interviews) | Luton & Dunstable, England | 31 Polish migrants | May to November 2011 |
| Sime, D. (2014). "'I think that Polish doctors are better': newly arrived migrant children and their parents׳ experiences and views of health services in Scotland." | Research paper | Mixed methods (Focus groups, Interviews, Diaries) | Scotland, urban and rural areas with a high proportion of new migrants | Recently arrived Eastern European children (and their parents). Polish, Lithuanian, Slovaks, Bulgarian, Romanian, Hungarian, Russian and Czech | Not stated |
| Sime, D. and R. Fox (2015). "Migrant Children, Social Capital and Access to Services Post-Migration: Transitions, Negotiations and Complex Agencies." | Research paper | Mixed methods (Focus groups, Interviews, Diaries) | Scotland, urban and rural areas with a high proportion of new migrants | Recently arrived Eastern European children (and their parents). Polish, Lithuanian, Slovaks, Bulgarian, Romanian, Hungarian, Russian and Czech | Not stated |
| Spencer, S., et al. (2007). "Migrants’ lives beyond the workplace." The experiences of central and east Europeans in the UK. York: Joseph Rowntree Foundation. | Report | Mixed methods (survey, interviews) | Not stated (UK) | 241 survey respondents and 43 Interviews. Czech, Slovak, Lithuanian and Polish. Comparison group – Ukrainian and Bulgarian. | Survey March to April 2004; Interviews November 2004 to February 2005 |
| Teshome, H. and J. Day (2015). "Health visitors' perceptions of barriers to health and wellbeing in European migrant families: The Journal of the Health Visitors' Association." | Research paper | Qualitative (Interviews) | Merseyside, England | 8 Health Visitors | 2012 |
| Turnbull et al., (2019) A conceptual model of urgent care sense-making and help-seeking: a qualitative interview study of urgent care users in England | Research Paper | Qualitative - longitudinal semi-structured interviews | Four counties in the South of England. | Participants from CEE Central Europe (18 at first interview, 12 at second) | September 2016 to July 2017 and June 2017 to November 2018 |
| Warren, K., et al. (2010). "Compilation of a local profile of immigration using GP registration data." | Research paper | Other - epidemiological profile | Walsall, West Midlands, England | EU Accession countries (Poland, Slovakia, Czech republic) | 2000 to 2008 depending of the different data sources |
| Zawacki, S., (2019) A Sense of Injustice: Experiences of the Central and Eastern European Roma in Accessing UK Health and Public Services | Thesis | Qualitative (Interviews) | London and Luton | Poland, Romania, Slovakia, Bulgaria - Interviews (27), Immigration focus group (9), Professional and Community worker interviews (10 - [Poland, UK, Ireland, Africa]) | 2015 to 2018 |
