# Supplementary material for: The perceptions of general practice among Central and Eastern Europeans in the United Kingdom: A systematic scoping review
Source: Health Expect. 2022 Jan 19;25(5):2107–23. doi: 10.1111/hex.13433 (PMC9615075; doi:10.1111/hex.13433)
Supplement: Supplementary file 4 — Supporting information. [file HEX-25--s004.docx]

## **Appendix D. Critical appraisal of publications included within the review**

**Critical appraisal scoring of individual studies**

The following tables detail critical appraisal ‘scores’ for each study included within the review. For each category, a score of ‘1’ indicates fulfilment of a category, while a score of ‘0’ indicated non-fulfilment. The potential maximum score for each measure (Max) is denoted in the first line, second column for each table.

A ‘traffic light summary’ has been incorporated with critical appraisal scores for individual publications’ colour coded to indicate whether a score is in the highest (green), middle (amber), or lowest (red) third of the score range for the critical appraisal measure used.

**Traffic light summary of critical appraisal scores**

| **Critical appraisal tool** | **Publications (n) in each critical appraisal score category** | | | **Total** |
| --- | --- | --- | --- | --- |
|  | Green (Highest 3rd) | Amber  (Middle 3rd) | Red  (Lowest 3rd) |  |
| Qualitative studies critically appraised using the CASP Qualitative Checklist | 30 | 6 | 1 | **37** |
| Analytical Cross Sectional studies critically appraised using the JBI Critical Appraisal Checklist for Analytical Cross Sectional Studies | 1 | 0 | 0 | **1** |
| Case series critically appraised using the JBI Critical Appraisal Checklist for Case Series | 2 | 2 | 0 | **4** |
| Mixed Method (Qualitative/Quantitative) studies critically appraised using the McGill University Mixed Methods Appraisal | 6 | 0 | 0 | **6** |
| Survey based studies critically appraised using the Centre for Evidence Based Medicine (CEBM) Survey appraisal tool | 2 | 3 | 2 | **7** |
| Cohort Studies critically appraised using the CASP Critical Appraisal tool for Cohort Studies | 2 | 1 | 0 | **3** |
| Case Report critically appraised using the JBI Critical Appraisal tool for Case Reports | 2 | 0 | 0 | **2** |
| Publications not meeting criteria for critical appraisal | 5 | 0 | 0 | **5** |
| **Cumulative Total** | **50** | **12** | **3** | **65** |

### **Qualitative studies critically appraised using the CASP Qualitative Checklist**

|  | **Score (Max 10)** | **1. Was there a clear statement of the aims of the research?** | **2. Is a qualitative methodology appropriate?** | **3. Was the research design appropriate to address the aims of the research?** | **4. Was the recruitment strategy appropriate to the aims of the research?** | **5. Was the data collected in a way that addressed the research issue?** | **6. Has the relationship between researcher and participants been adequately considered?** | **7. Have ethical issues been taken into consideration?** | **8. Was the data analysis sufficiently rigorous?** | **9. Is there a clear statement of findings?** | **10. How valuable is the research?** |
| --- | --- | --- | --- | --- | --- | --- | --- | --- | --- | --- | --- |
| Bell, S., et al. (2019). | 9 | 1 | 1 | 1 | 1 | 1 | 0 | 1 | 1 | 1 | 1 |
| Bell, S., et al. (2020). | 9 | 1 | 1 | 1 | 1 | 1 | 0 | 1 | 1 | 1 | 1 |
| Bray, J. K., et al. | 8 | 1 | 0 | 1 | 1 | 1 | 0 | 1 | 1 | 1 | 1 |
| Cleland, J. A., et al. (2012). | 9 | 1 | 1 | 1 | 1 | 1 | 0 | 1 | 1 | 1 | 1 |
| Close, C. M., et al. (2018). | 9 | 1 | 1 | 1 | 1 | 1 | 0 | 1 | 1 | 1 | 1 |
| Collis, A., et al. (2010). | 5 | 1 | 1 | 1 | 0 | 1 | 0 | 0 | 0 | 0 | 1 |
| Condon, L. J. and D. Salmon (2015). | 10 | 1 | 1 | 1 | 1 | 1 | 1 | 1 | 1 | 1 | 1 |
| Condon, L. J. and S. McClean (2017). | 8 | 1 | 1 | 1 | 1 | 1 | 0 | 1 | 1 | 1 | 0 |
| Cook, J., et al. (2012). | 6 | 1 | 1 | 1 | 1 | 0 | 0 | 0 | 0 | 1 | 1 |
| Crowther, S. and A. Lau (2019). | 9 | 1 | 1 | 1 | 1 | 1 | 0 | 1 | 1 | 1 | 1 |
| Gorman, D. and L. Porteous (2018). | 8 | 1 | 1 | 1 | 1 | 1 | 0 | 0 | 1 | 1 | 1 |
| Gorman, D. R., et al. (2019). | 8 | 1 | 1 | 1 | 1 | 1 | 0 | 0 | 1 | 1 | 1 |
| Jackowska, M., et al. (2012). | 9 | 1 | 1 | 1 | 1 | 1 | 0 | 1 | 1 | 1 | 1 |
| Jackson, C., et al. (2017). | 9 | 1 | 1 | 1 | 1 | 1 | 0 | 1 | 1 | 1 | 1 |
| Kouvonen et al. (2014) | 1 | 0 | 1 | 0 | 0 | 0 | 0 | 0 | 0 | 0 | 0 |
| Lehane, D., et al. (2020). | 7 | 1 | 1 | 1 | 0 | 1 | 0 | 0 | 1 | 1 | 1 |
| Lindenmeyer, A., et al. (2016a) | 6 | 0 | 1 | 1 | 1 | 1 | 0 | 0 | 0 | 1 | 1 |
| Lindenmeyer A, (2016b). | 9 | 1 | 1 | 1 | 1 | 1 | 0 | 1 | 1 | 1 | 1 |
| MacKichan F, et al. (2017) | 9 | 1 | 1 | 1 | 1 | 1 | 0 | 1 | 1 | 1 | 1 |
| Madden, H., et al. (2017). | 10 | 1 | 1 | 1 | 1 | 1 | 1 | 1 | 1 | 1 | 1 |
| Main, I. (2016). | 8 | 1 | 1 | 1 | 0 | 1 | 1 | 1 | 0 | 1 | 1 |
| Mills, K. and T. Knight (2010). | 6 | 0 | 1 | 1 | 0 | 1 | 0 | 0 | 1 | 1 | 1 |
| Osipovič, D. (2013) | 8 | 1 | 1 | 1 | 1 | 1 | 1 | 0 | 0 | 1 | 1 |
| Patel, H., et al. (2020). | 9 | 1 | 1 | 1 | 1 | 1 | 0 | 1 | 1 | 1 | 1 |
| Phillimore, J. (2011). | 6 | 0 | 1 | 1 | 1 | 1 | 0 | 0 | 0 | 1 | 1 |
| Pope, C., et al. (2019). | 8 | 0 | 1 | 1 | 1 | 1 | 0 | 1 | 1 | 1 | 1 |
| Richards, J., et al. (2014). | 8 | 1 | 1 | 1 | 1 | 1 | 0 | 0 | 1 | 1 | 1 |
| Selkirk, M., et al. (2012). | 10 | 1 | 1 | 1 | 1 | 1 | 1 | 1 | 1 | 1 | 1 |
| Sharp, C. and G. Randhawa (2015). | 9 | 1 | 1 | 1 | 1 | 1 | 0 | 1 | 1 | 1 | 1 |
| Sharp, C. and G. Randhawa (2016). | 9 | 1 | 1 | 1 | 1 | 1 | 0 | 1 | 1 | 1 | 1 |
| Sime, D. (2014). | 7 | 1 | 1 | 1 | 0 | 1 | 0 | 0 | 1 | 1 | 1 |
| Sime, D. and R. Fox (2015). | 7 | 1 | 1 | 1 | 0 | 1 | 0 | 0 | 1 | 1 | 1 |
| Spencer, S., et al. (2007). | 7 | 1 | 1 | 1 | 1 | 1 | 0 | 0 | 0 | 1 | 1 |
| Teshome, H. and Day J. (2015). | 9 | 1 | 1 | 1 | 1 | 1 | 0 | 1 | 1 | 1 | 1 |
| Turnbull et al. (2019). | 9 | 1 | 1 | 1 | 1 | 1 | 0 | 1 | 1 | 1 | 1 |
| Warren, K., et al. (2010). | 6 | 1 | 0 | 1 | 1 | 1 | 0 | 0 | 0 | 1 | 1 |
| Zawacki, S., (2019) | 10 | 1 | 1 | 1 | 1 | 1 | 1 | 1 | 1 | 1 | 1 |

### **Analytical Cross Sectional studies critically appraised using the JBI Critical Appraisal Checklist for Analytical Cross Sectional Studies**

|  | **Score (Max 8)** | **1. Were the criteria for inclusion in the sample clearly defined?** | **2. Were the study subjects and the setting described in detail?** | **3. Was the exposure measured in a valid and reliable way?** | **4. Were objective, standard criteria used for measurement of the condition?** | **5. Were confounding factors identified?** | **6. Were strategies to deal with confounding factors stated?** | **7. Were the outcomes measured in a valid and reliable way?** | **8. Was appropriate statistical analysis used?** |
| --- | --- | --- | --- | --- | --- | --- | --- | --- | --- |
| Platt, L., et al. (2011). | 8 | 1 | 1 | 1 | 1 | 1 | 1 | 1 | 1 |

### **Case series critically appraised using the JBI Critical Appraisal Checklist for Case Serie**s

|  | **Score (Max 10)** | **1. Were there clear criteria for inclusion in the case series?** | **2. Was the condition measured in a standard, reliable way for all participants included in the case series?** | **3. Were valid methods used for identification of the condition for all participants included in the case series?** | **4. Did the case series have consecutive inclusion of participants?** | **5. Did the case series have complete inclusion of participants?** | **6. Was there clear reporting of the demographics of the participants in the study?** | **7. Was there clear reporting of clinical information of the participants?** | **8. Were the outcomes or follow up results of cases clearly reported?** | **9. Was there clear reporting of the presenting site(s)/clinic(s) demographic information?** | **10. Was statistical analysis appropriate?** |
| --- | --- | --- | --- | --- | --- | --- | --- | --- | --- | --- | --- |
| Brawley, D., et al. (2013) | 8 | 1 | 1 | 1 | 1 | 1 | 1 | 1 | 1 | 0 | 0 |
| Gill, G. (2009). | 4 | 1 | 0 | 0 | 0 | 0 | 1 | 1 | 1 | 0 | 0 |
| Gorman, D., et al. (2018). | 10 | 1 | 1 | 1 | 1 | 1 | 1 | 1 | 1 | 1 | 1 |
| O’Neill, B., (2011) | 6 | 1 | 1 | 1 | 1 | 0 | 1 | 0 | 0 | 1 | 0 |

### **Mixed Method (Qualitative/Quantitative) studies critically appraised using the McGill University Mixed Methods Appraisal**

N.B. Table incorporates 2 sections.

|  | **Score (Max 17)** | **S1. Are there clear research questions?** | **S2. Do the collected data allow to address the research questions?** | **1.1. Is the qualitative approach appropriate to answer the research question?** | **1.2. Are the qualitative data collection methods adequate to address the research question?** | **1.3. Are the findings adequately derived from the data?** | **1.4. Is the interpretation of results sufficiently substantiated by data?** | **1.5. Is there coherence between qualitative data sources, collection, analysis and interpretation?** |
| --- | --- | --- | --- | --- | --- | --- | --- | --- |
| Blake, H., et al. (2018). | 14 | 1 | 1 | 1 | 1 | 1 | 1 | 1 |
| Chojnacki, S. (2020) | 14 | 1 | 1 | 1 | 0 | 1 | 1 | 1 |
| Evans, A. R., et al. (2009). | 16 | 1 | 1 | 1 | 1 | 1 | 1 | 1 |
| Goodwin, R., et al. (2013). | 13 | 1 | 1 | 1 | 1 | 1 | 1 | 1 |
| Khalid, T., (2018) | 15 | 1 | 1 | 1 | 1 | 1 | 1 | 1 |
| Penn, R., (2008) | 14 | 1 | 1 | 1 | 1 | 1 | 1 | 1 |

|  | **4.1. Is the sampling strategy relevant to address the research question?** | **4.2. Is the sample representative of the target population?** | **4.3. Are the measurements appropriate?** | **4.4. Is the risk of nonresponse bias low?** | **4.5. Is the statistical analysis appropriate to answer the research question?** | **5.1. Is there an adequate rationale for using a mixed methods design to address the research question?** | **5.2. Are the different components of the study effectively integrated to answer the research question?** | **5.3. Are the outputs of the integration of qualitative and quantitative components adequately interpreted?** | **5.4. Are divergences and inconsistencies between quantitative and qualitative results adequately addressed?** | **5.5. Do the different components of the study adhere to the quality criteria of each tradition of the methods involved?** |
| --- | --- | --- | --- | --- | --- | --- | --- | --- | --- | --- |
| Blake, H., et al. (2018). | 1 | 0 | 1 | 0 | 1 | 1 | 1 | 1 | 0 | 1 |
| Chojnacki, S. (2020) | 1 | 1 | 1 | 0 | 1 | 1 | 1 | 1 | 0 | 1 |
| Evans, A. R., et al. (2009). | 1 | 1 | 1 | 0 | 1 | 1 | 1 | 1 | 1 | 1 |
| Goodwin, R., et al. (2013). | 1 | 0 | 1 | 0 | 1 | 1 | 1 | 1 | 0 | 0 |
| Khalid, T., (2018) | 1 | 1 | 1 | 0 | 1 | 1 | 1 | 1 | 0 | 1 |
| Penn, R., (2008) | 1 | 0 | 1 | 0 | 1 | 1 | 1 | 1 | 0 | 1 |

### **Survey based studies critically appraised using the Centre for Evidence Based Medicine (CEBM) Survey appraisal tool**

|  | **Score (Max 12)** | **1. Did the study address a clearly focused question / issue?** | **2. Is the research method (study design) appropriate for answering the research question?** | **3. Is the method of selection of the subjects (employees, teams, divisions, organizations) clearly described?** | **4. Could the way the sample was obtained introduce (selection) bias?** | **5. Was the sample of subjects representative with regard to the population to which the findings will be referred?** | **6. Was the sample size based on pre-study considerations of statistical power?** | **7. Was a satisfactory response rate achieved?** | **8. Are the measurements (questionnaires) likely to be valid and reliable?** | **9. Was the statistical significance assessed?** | **10. Are confidence intervals given for the main results?** | **11. Could there be confounding factors that haven’t been accounted for?** | **12. Can the results be applied to your organization?** |
| --- | --- | --- | --- | --- | --- | --- | --- | --- | --- | --- | --- | --- | --- |
| Burns et al. (2011). | 9 | 1 | 1 | 1 | 0 | 1 | 0 | 1 | 1 | 1 | 1 | 0 | 1 |
| Collinson, S. and R. Ward (2010). | 3 | 0 | 1 | 1 | 0 | 0 | 0 | 0 | 0 | 0 | 0 | 0 | 1 |
| Evans, A. R., et al. (2011). | 8 | 1 | 1 | 1 | 0 | 1 | 0 | 1 | 1 | 1 | 0 | 0 | 1 |
| Evans, A., et al. (2010). | 5 | 1 | 1 | 1 | 0 | 1 | 0 | 0 | 0 | 0 | 0 | 0 | 1 |
| Gondek, D. and J. B. Kirkbride (2018). | 9 | 1 | 1 | 1 | 0 | 1 | 0 | 1 | 1 | 1 | 1 | 0 | 1 |
| Gorman, D. R., et al. (2020). | 7 | 1 | 1 | 1 | 0 | 1 | 0 | 0 | 1 | 1 | 0 | 0 | 1 |
| Lincolnshire County Council, (2013) | 1 | 0 | 0 | 0 | 0 | 0 | 0 | 0 | 0 | 0 | 0 | 0 | 1 |

### **Cohort Studies critically appraised using the CASP Critical Appraisal tool for Cohort Studies**

|  | **Score (Max 12)** | **1. Did the study address a clearly focused question / issue?** | **2. Was the cohort recruited in an acceptable way?** | **3. Was the exposure accurately measured to minimise bias?** | **4. Was the outcome accurately measured to minimise bias?** | **5. (a) Have the authors identified all important confounding factors? (b) Have they taken account of the confounding factors in the design and/or analysis?** | **6. (a) Was the follow up of subjects complete enough? (b) Was the follow up of subjects long enough?** | **7. What are the results of this study?** | **8. How precise are the results? (CIs)** | **9. Do you believe the results?** | **10. Can the results be applied to the local population?** | **11. Do the results of this study fit with other available evidence?** | **12. What are the implications of this study for practice?** |
| --- | --- | --- | --- | --- | --- | --- | --- | --- | --- | --- | --- | --- | --- |
| Bielecki, K., et al. (2019). | 11 | 1 | 1 | 1 | 1 | 0 | 1 | 1 | 1 | 1 | 1 | 1 | 1 |
| Ignaszak-Szczepaniak, M., et al. (2009). | 8 | 1 | 1 | 1 | 1 | 0 | 0 | 1 | 0 | 1 | 0 | 1 | 1 |
| Pollock., et al. (2007) | 9 | 1 | 1 | 0 | 1 | 1 | 1 | 1 | 0 | 1 | 1 | 1 | 0 |

### **Case Report critically appraised using the JBI Critical Appraisal tool for Case Reports**

|  | **Score (Max 8)** | **1. Were patient’s demographic characteristics clearly described?** | **2. Was the patient’s history clearly described and presented as a timeline?** | **3. Was the current clinical condition of the patient on presentation clearly described?** | **4. Were diagnostic tests or assessment methods and the results clearly described?** | **5. Was the intervention(s) or treatment procedure(s) clearly described?** | **6. Was the post-intervention clinical condition clearly described?** | **7. Were adverse events (harms) or unanticipated events identified and described?** | **8. Does the case report provide takeaway lessons?** |
| --- | --- | --- | --- | --- | --- | --- | --- | --- | --- |
| Hodson, N. and R. Glennerster (2020). | 8 | 1 | 1 | 1 | 1 | 1 | 1 | 1 | 1 |
| Hodgekiss, C.H.E. and Shipman, A.R., (2010). | 7 | 1 | 1 | 1 | 1 | 1 | 0 | 1 | 1 |

### **Publications not meeting criteria for critical appraisal**

|  | **Study type** | **Publication type** | **Comment** |
| --- | --- | --- | --- |
| Bosqui, T., et al. (2019). | Record linkage study | Research paper | No clear appraisal design |
| Fitzgerald, I. and R. Smoczynski (2017). | Policy document | Report (article providing a reflection on a topic, not peer reviewed) | Article providing a reflection on a topic, not peer reviewed |
| Ford, A., et al. (2013 | Unclear | Online magazine article | Unclear study design |
| Giuntella, O., et al. (2018 | Health economics | Research paper | Health economics analysis |
| Leaman, A. M., et al. (2006 | Audit | Report | Audit |
